# COVID-19 Genetic and Environmental Risk Factors: A Look at the Evidence

**DOI:** 10.3389/fphar.2020.579415

**Published:** 2020-10-07

**Authors:** Hana Abdelzaher, Basma M. Saleh, Hebatalla A. Ismail, Marwa Hafiz, Macy Abou Gabal, Miranda Mahmoud, Sarah Hashish, Rana M. Abdel Gawad, Rami Y. Gharieb, Anwar Abdelnaser

**Affiliations:** School of Science and Engineering, Institute of Global Health and Human Ecology, The American University in Cairo, Cairo, Egypt

**Keywords:** COVID-19, SARS-CoV-2, infection, risk-factors, molecular predisposition, environmental and occupational exposure

## Abstract

The Covid-19 pandemic is with no doubt the biggest health crisis of the 21^st^ century. The disease is caused by a virus of the *Coronaviridae* family and is closely related to the virus responsible for the severe acute respiratory Syndrome (SARS). Since December 2019, the virus has continued to spread way beyond the location of the first recorded cases (Wuhan, China). As of now, over 5 million cases have been diagnosed with the disease worldwide and over 300 thousand have died. COVID-19 patients suffer from respiratory symptoms that can rapidly turn into potentially fatal acute respiratory distress syndrome (ARDS) in a portion of patients. Although many drugs and vaccines are currently under clinical trials, there is no currently approved treatment or vaccine. It is therefore critical to correctly identify risk factors that lead to the exacerbation of symptoms in highly susceptible groups. Groups that are at high risk include those aged 55 or older especially those with underlying conditions such as cardiovascular diseases. Certain ethnicities such as African-Americans have been found to be at a higher risk and males seem to be higher both in numbers as well as severity of cases. It is hypothesized that these groups are at risk as their molecular landscape is more permissive of viral infection and growth. Different occupations, especially those related to health-care as well as populations that do not cultivate a mask-wearing culture are at higher risk due to environmental exposure. In this article, we examine the evidence regarding different groups that are more sensitive to the disease and review hypotheses pertaining to COVID-19 infection and prognosis. Risk factors that can be related to the molecular landscape of COVID-19 infection as well as those related to environmental and occupational conditions are discussed.

## Introduction

Coronavirus is named as the corona due to the presence of crown-like spikes on the outside of the virus (Ouassou et al., 2020). Coronaviruses (CoVs) are enveloped, non-segmented viruses with a definite, positive sense single-stranded RNA genome (Arabi et al., 2020; Chen J. et al., 2020). This family of viruses has always been considered a family of nonfatal pathogens broadly distributed in humans and other mammals. Usually, they were responsible for causing 15% of common colds cases (Yi et al., 2020). In the last two decades, severe acute respiratory syndrome-related coronavirus (SARS-CoV), which caused a disease of severe acute respiratory syndrome (SARS) appeared in China with a 10% mortality rate (Fauci et al., 2020). Another CoV, Middle East respiratory syndrome-related coronavirus (MERS-CoV) spread in Saudi Arabia with a mortality rate of 37% in the year of 2012 (Huang et al., 2020).

The coronaviruses already identified till now might only be the tip of the iceberg, with potentially more novel and severe zoonotic events to be revealed (Wang C. et al., 2020a). In this review, we summarize various trends of the spread of COVID-19, the genetic landscape of the SARS-CoV-2 virus and perform an in-depth analysis of current evidence pertaining to factors affecting its virulence and transmissibility.

### The SARS-COV-2 Timeline

**Figure 1:**
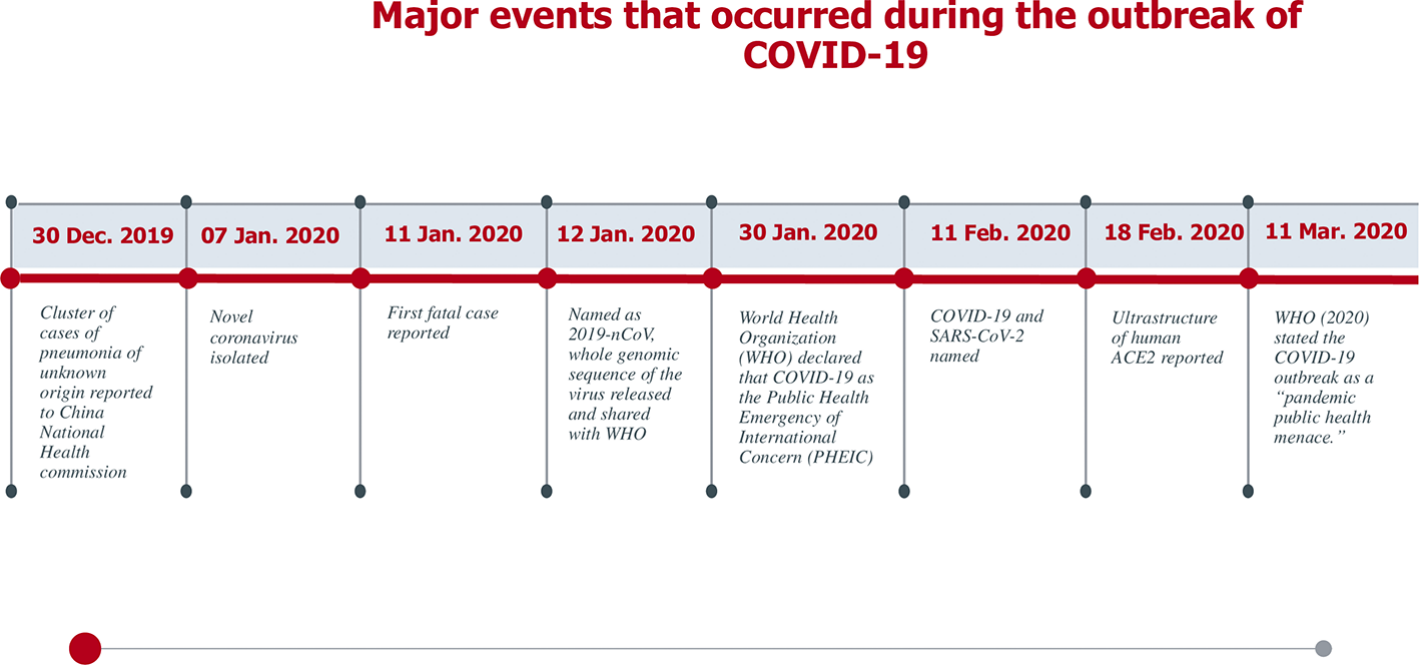
A timeline of major events that occurred during the COVID-19 outbreak.

**Figure 2:**
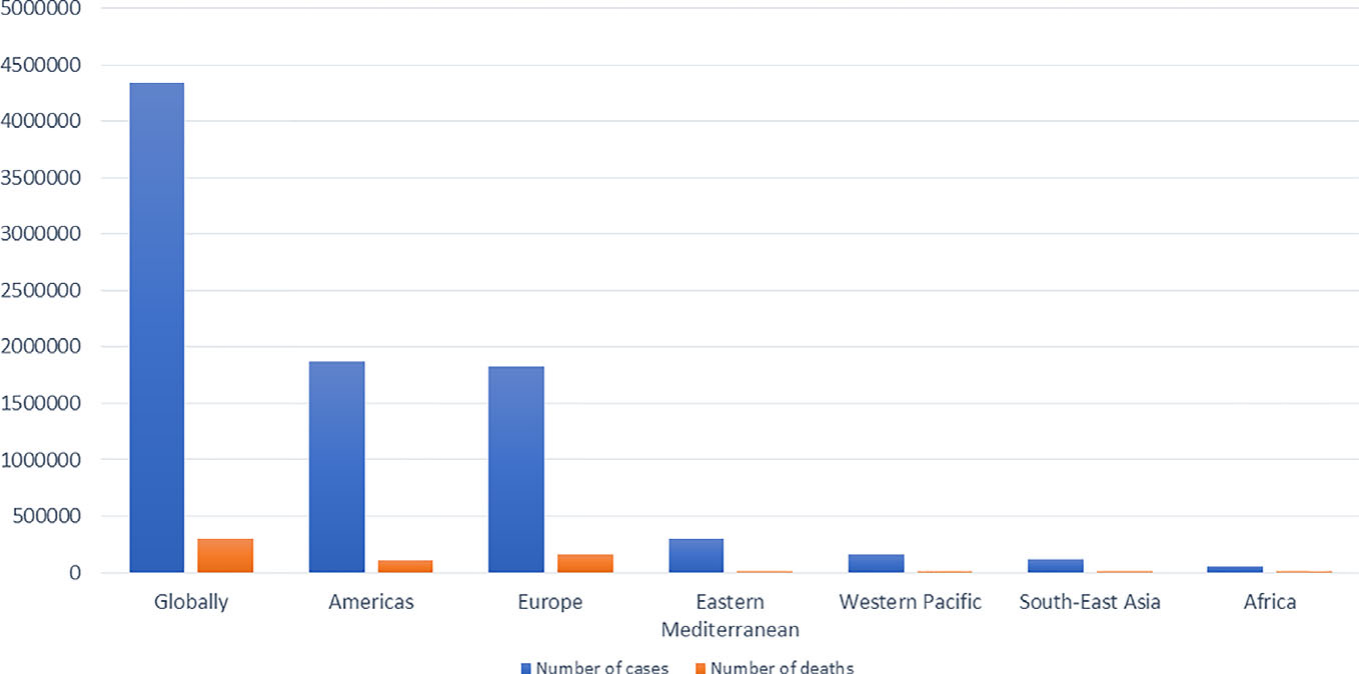
The current situation as reported by the WHO on 15th of May, 2020.

Around January 25, 2020, an outbreak of a series of pneumonia cases caused by a novel viral disease caused the Chinese government to issue stay-at-home warnings (Yi et al., 2020). Sequencing of the viral genome was obtained from bronchoalveolar lavage fluids and throat swabs of five hospitalized infected patients with pneumonia, which revealed the presence of a novel β-CoV strain from all of them to be then named as “SARS-CoV-2” by the International Virus Classification Commission (IVCC) (Yi et al., 2020). The outbreak started in Wuhan, China, and quickly spread throughout the entire country and to more than 100 other countries all over the world (Wang et al., 2020a) causing more than 3,750,000 infected cases and more than 270,000 deaths (France press, 14 May, 2020). A number of milestones in the trajectory of SARS-CoV-2 is shown in **Figure 1** (Peng et al., 2020; Yi et al., 2020). The current situation globally as reported by the World Health Organization (WHO) on the 15^th^ of May 2020 is shown in **Figure 2** (Livingston et al., 2020).

### COVID-19 Clinical Picture, Diagnosis, and Treatment Protocol

Symptoms resulting from 2019-nCoV infection at the prodromal phase, including fever, malaise, diarrhea, and dry cough, are nonspecific (Huang et al., 2020) although they are the most common symptoms (Repici et al., 2020). Upper respiratory symptoms were unusual findings unlike the other human coronavirus infections (Repici et al., 2020). In a study reporting the clinical findings of the first 41 COVID-19 patients, six common laboratory findings were observed including lymphopenia and the most characteristic radiological finding which is peripheral bilateral ground-glass opacity or consolidation in chest CT scans (Fauci et al., 2020). These clinical findings were much helpful in early detection of infected cases, in contrast to other ongoing common cold or influenza circulating among the population. Exposure history to the Huanan Seafood Wholesale market was a significant clue for a while, but lost its value as more secondary and tertiary cases were developed (Huang et al., 2020). It is now known that the disease is a contagious acute respiratory disease, transmitted either by direct contact with the carrier-host, their infected droplets or with other respiratory secretions (Patel et al., 2020).

One of the reasons contributing to the gravity of COVID-19 is its ability to rapidly spread during the latent period, since the incubation period would usually last from 1 to 14 days (Guo et al., 2020). The U.S. Centers for Disease Control stated that the common symptoms shown among COVID-19 patients were fever, cough, and difficulty with breathing. In mild cases, the symptoms would include mild fever, sore throat, headache, malaise, and dry cough. Later, the symptoms would be aggravated with moderate illness when dyspnea begins to appear. In severe illness, the patients would have fever, tachypnea that would reach more than 30 breaths per minute and respiratory distress. In more unfortunate conditions, the patients in critical statuses would be suffering from either an acute respiratory distress syndrome or a septic shock which would increase the risk of mortality (Cascella et al., 2020). Quantitative Real-time polymerase chain reaction remains the reference method for diagnosis of SARS-CoV-2 infection, serological assays for antibody detection may also be used yet they display varying sensitivity according to the time of infection (Mathuria and Yadav, 2020). Radiographic examinations could be an aiding tool to assess the situation, but never a diagnostic one. This is due to the fact that the sensitivity of radiography is limited, and many patients do not demonstrate any significant findings in their CT scans. However, the shared distinctive radiographic features in some cases are ground-glass opacity and bilateral patchy shadowing on chest CT (Cascella et al., 2020; Guo et al., 2020; Hassan et al., 2020; Wang et al., 2020a).

Last January, the WHO published a guideline based on the previous protocols followed in the earlier human Coronaviruses pandemics, discussing the early monitoring and supportive paradigms along with the necessity of providing an extracorporeal membrane oxygenation therapy in more severe cases (Cascella et al., 2020). Several studies suggest the administration of antiviral drugs, such as Remdesivir, Ribavirin or Abidor. It was revealed that the use of Remdesivir inhibit the infection of RNA viruses, yet it is important to add that the consumption of three or more drugs concurrently is unfavorable (Wang et al., 2020a). On May 1, 2020, The US Food and Drug Administration (FDA) issued Emergency Use Authorization (EUA) of Remdsivir to allow emergency use of the agent for severe COVID-19 (confirmed or suspected) in hospitalized adults and children (Commissioner O of the, 2020a).

Chloroquine is a treatment for malaria, and it was reported to have an effective antiviral action against SARS-CoV and MERS-CoV *in vitro*. For this reason, it was used with COVID-19 cases as it was suggested that it prevents the aggravation of the disease, and it causes viral inhibition (Ahn et al., 2020). However, a recently retracted study on over 96000 COVID-19 patients led to a confusion. Mehra et al. concluded that they have not seen any benefit from the administration of chloroquine nor hydrochloroquine when used to treat COVID-19. On the contrary, their use even increased the incidence of ventricular arrhythmias (Mehra et al., 2020). Lastly, organ support is vital to prevent the risk of organ damage and collapse with patients suffering from comorbidities (Hassan et al., 2020). Due to the varying symptoms of COVID-19, not all cases require ventilation or intubation. The severe complications that necessitate an oxygen therapy intervention are acute hypoxemic respiratory failure or acute respiratory distress syndrome (ARDS). Some studies advise ventilation while positioning the patient in the prone position during the early phase of the disease. This method will reduce the mortality with severe ARDS cases (Guérin et al., 2013). There is not enough evidence that a ventilation mode is more efficient than another, yet the utilization of high frequency oscillatory ventilation is unfavorable because it may lead to aerosol generation. Intubation does not guarantee the cure of COVID-19. For instance, last March, 19 out of 22 intubated patients died in Wuhan. It was suggested that this loss might be due to the delay in the decision making, since a previous study by Shoemaker et al. explained a close connection between preventing the accumulation of oxygen debt within 48 h and a higher chance of patients’ survival (Shoemaker et al., 1992; Meng et al., 2020).

### Properties of COVID-19 (Differences and Similarities Between COVID-19 and Other Viruses of the Coronaviridae Family)

Coronaviruses are considered the largest genera among the Nidovirales family (Fehr and Perlman, 2015). Throughout the years, there were seven strains of Human Coronaviruses detected worldwide. The 229E and NL63 are examples of the Alpha type, while HKU1, OC43, SARS, MERS, and the novel COVID-19 represent the Beta type (Ibrahim et al., 2020). These identified strains share some common characteristics; they are from 27 up to 32 kb long, positive-sense, contagious, and enveloped RNA viruses. Coupled with these criteria, it was recognized that they contain structural and non-structural proteins. Among these structural proteins is the spike protein (S) which is essential to facilitate the entry of the virus into the host cell by binding to angiotensin-converting enzyme 2 (ACE2) receptor. S protein is one of the key factors upon which the severity of the virulence is determined. In addition to the S protein, it was discovered that the nucleocapsid (N) protein forms a capsid in which the genome of the virus is packed, while the membrane (M) and envelope (E) proteins are responsible for the formation of the virus’ envelope. Finally, it was also revealed that some of the viruses may contain an envelope-associated hemagglutinin-esterase protein (HE). Whereas, the non-structural proteins (nsps) include nsp3, nsp5, and nsp12 (Li, 2016). In light of the careful studies concerning the strains’ protein sequence, we now have a better understanding of the emerging SARS-CoV-2 (Li, 2016; Ibrahim et al., 2020). Data suggest that SARS and other human coronaviruses were persistent in nature, utilizing animals as a reservoir, circulating, evolving their strains, and causing disease outbreaks in smaller scales for years (Graham and Baric, 2010).

Despite the fact that the vaccines are a convenient therapeutic solution, there was no vaccine developed to treat or prevent any of the coronaviruses discovered during the last decades. Several approaches like mRNA vaccines which are considered a new generation were carefully studied. Generally, mRNA vaccines are more preferable than conventional vaccines due to the higher immune response. Clinical trials are currently concentrating on developing an mRNA vaccine that would encode the S protein of COVID-19. Another approach is subunit vaccines. Subunit vaccines are extremely safe because they enhance the hosts’ immune response without introducing a viral component. For that reason, the “molecular clamp” is a trial to develop a polypeptide that would enhance the identification of antigens and boost the immunity against a wide range of enveloped viruses. Other modalities were focusing on DNA vaccines that would increase the activation of T cells, or antibody-dependent enhancement (ADE) which is a method to introduce virus-specific antibodies. *In vitro*, ADE tests were successful with MERS-CoV and SARS-CoV (Ahn et al., 2020). However, for the time being, the primary therapeutic line against the virus is isolation, symptomatic support, nutritious diet, and oxygen therapy.

## The COVID-19 Molecular Landscape

### Genome Characterization 

As stated earlier, six strains of coronaviruses have been detected until 2019 and are able to cause human infections (Cui et al., 2019), of which are four human coronaviruses, which are not highly infective and can only lead to mild respiratory infections, and these include HCoV-OC43, HCoV-229E, HCoV-NL63, and HCoVHKU1 (Cui et al., 2019). In contrast, the other two coronaviruses are SARS-CoV-1 (Zhong et al., 2003) and MERS-CoV (Zaki et al., 2012). In 2003, SARS-CoV-1 gave rise to more than 8000 cases, involving nearly 800 related deaths in 26 countries. Additionally, in 2012, MERS CoV has resulted in around 2500 cases, involving about 860 related deaths in Saudi Arabia (Cui et al., 2019).

Soon after China declared Covid-19 an outbreak, they started analyzing its genome sequences by isolating samples from 9 inpatients, eight of them had just visited the wet market in Wuhan. Bronchoalveolar lavage fluids had been collected from patients to perform second generation sequencing; and viral segments had been gathered using Sanger sequencing method for a full-length genome analysis (Lu et al., 2020). Results had shown that the sequence identity was above 99.98 % across the whole isolated complete genome in the collected samples indicating a new emergence of the virus into humans. Two partial isolated genomes (WH02 and WH19002) had been sequenced as well; as a result, they showed almost 100% similarity to the complete genome (Lu et al., 2020). Lu et al. concluded that the new SARS-CoV-2 is closely linked to bat-SL-CoVZC45 and another SARS-like beta-coronavirus of bat origin, bat-SL-CoVZXC21. In line with the scientific literature, these research findings support that SARS-CoV-2 has a bat origin and is 96% identical on the molecular level (Lu et al., 2020). Findings also revealed that SARS-CoV-2 strains were less molecularly identical to SARS-CoV-1 (around 79%) and MERS-CoV (around only 50%). Most encoded proteins showed significant identity between SARS-CoV-2 and bat-derived coronaviruses, except for the spike protein that showed around 80% and protein 13 was only identical by 73.2% (Lu et al., 2020).

Phylogenetic analysis of SARS-CoV-2 and related viruses of the genus beta-coronaviruses supports that SARS-CoV-2 is a novel beta-coronavirus that belongs to the subgenus Sarbecovirus. Additionally, SARS-CoV-2 is distinct from SARS-CoV-1 in phylogeny. The phylogenetic reconstruction of SARS-CoV-2 based on genome analysis might be complicated, as a recent study has shown that there might be an evolutionary recombination and selection pattern between CoVs from distinct hosts, meaning that cross-species infections might have led to the emergence of SARS-CoV-2 (Li X. et al., 2020). The origin of SARS-CoV-2 and its genome characterization might have evolved from ancestral recombinations between viruses that attack bats and pangolins along with purifying selection patterns in different host species (Li X. et al., 2020).

### Molecular Mechanism of COVID-19 Infection

**Figure 3:**
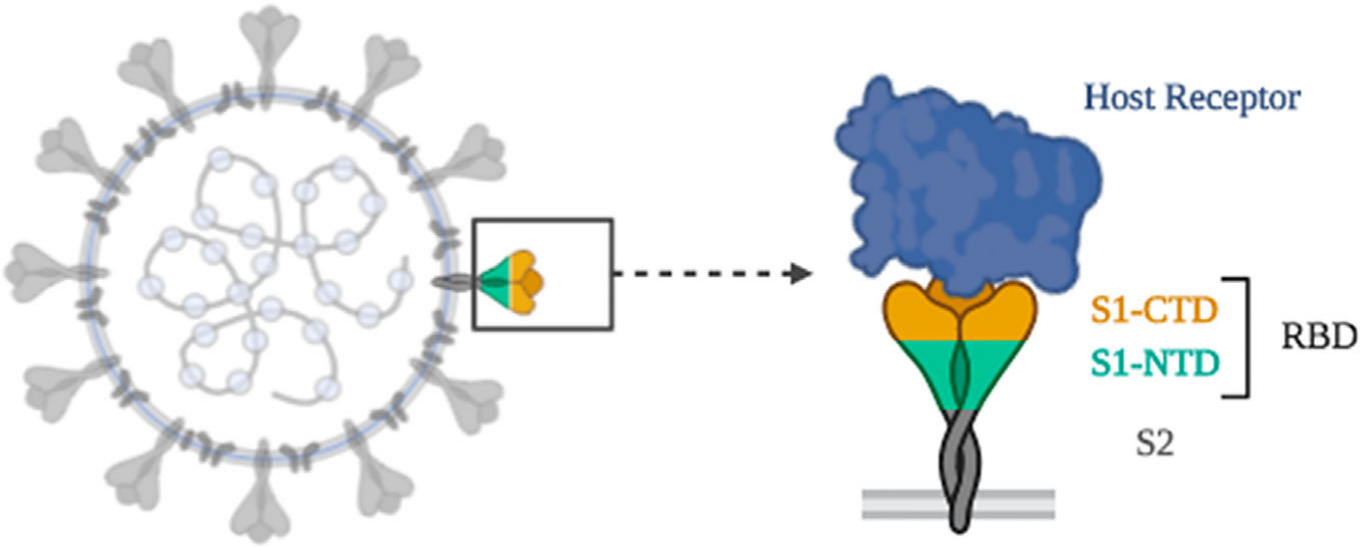
Schematic of receptor-binding mechanism of SARS-CoV-2.

Coronaviruses make use of the S glycoprotein, a spike protein on the envelope of the coronavirus surface to get attached to host cells, in mediating the fusion of host cell membrane and viral membrane during pathogenicity (Li, 2016). S glycoprotein is composed of two regions, S1 and S2; S1 is for receptor binding and S2 is for membrane fusion. The S1 region involves N-terminal domain and three C-terminal domains, CTD1, CTD2, and CTD3 (Gui et al., 2017). The RBD of SARS-CoV-1 is placed in CTD1 of the S1 region. Therefore, for SARS-CoV-1 to attach to human host cells, RBD and angiotensin-converting enzyme II interaction should first get activated (**Figure 3**) (Song et al., 2018). It is expected that SARS-CoV-2 exploits the same mechanism as SARS-CoV-1 to infect human cells due to the high homology between the two viruses (Wan et al., 2020).

Studies have shown that predicted SARS-CoV-2 RBD and angiotensin-converting enzyme II complex is in close proximity to the determined RBD-ACE2 complex structure of SARS-CoV-1. Additionally, the RBD of SARS-CoV-2 binds to the same position of the ACE2 receptor as SARS-CoV-1 (He et al., 2020). MD simulation was used to get insights into the ways the RBD-ACE2 complex formed in both SARS-CoV-1 and SARS-CoV-2. SARS-CoV-2 has a lower binding free energy (-50.43 kcal/mol) than SARS-CoV-1 (-36.75 kcal/mol); meaning that SARS-CoV-2 binds ACE2 with greater affinity than SARS-CoV-1. These results show that SARS-CoV-2 is more pathogenic than its predecessor.

Researchers have been interested in studying and understanding the interplay between ACE2 and the S protein of SARS-CoV-2 so they can come up with a potential therapeutic approach since this interaction is considered a prerequisite of Covid-19. The renin–angiotensin–aldosterone system (RAAS) is a distinctive cascade of vasoactive peptides assembling key processes in human physiology (Vaduganathan et al., 2020). SARS-like CoVs intermingle with RAAS through ACE2 that counters RAAS activation. Therefore, researchers have been trying to observe the effect of RAAS inhibitors on the expression of ACE2 that may result in the ongoing COVID-19 pandemic. Experimental animal models have shown inconsistent outcomes in regards to ARBs on ACE2; some studies showed an increase in the mRNA expression of ACE2 due to ARBs, others showed that ARBs had no effect on ACE2 expression (Vaduganathan et al., 2020). With respect to the few studies conducted on humans, one study demonstrated that the intravenous administration of ACE inhibitors in coronary artery disease patients did not impact the production of angiotensin-(1–7), a finding that raises questions whether ACE inhibitors have any direct effects on ACE2-directed angiotensin II metabolism (Campbell et al., 2004)

Another cross sectional study involved patients with cardiovascular diseases showed that the activity of plasma ACE2 among patients who had taken ACE inhibitors or ARBs was not higher compared to untreated patients (Vaduganathan et al., 2020). These observations might be explained through looking into the different effects imposed by ACE inhibitors and ARBs on angiotensin II; their active sites are distinct, and the ACE inhibitors do not directly influence the ACE2 expression (Rice et al., 2004).

Additionally, a previous treatment involving ACE inhibitors was correlated with an elevation in the intestinal mRNA of ACE2 in one of the studies; however, this finding was not shown with ARBs; no data are available in regards to the effects of RAAS inhibitors on the lung’s ACE2 expression (Vuille-dit-Bille et al., 2015). Thus, no accurate data are available to decide whether these outcomes translate to humans, and further research is needed to investigate the role of RAAS inhibitors with COVID-19 patients; also, other receptors might be involved in the entry of SARS-CoV-2 into human host cells.

### Spike Mutations and the Emergence of a More Communicable Form of SARS-CoV-2

**Figure 4:**
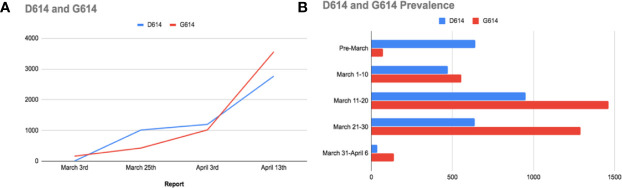
**(A)** A comparison between the D614 and G614 spread worldwide. **(B)** Bar charts representing the prevalence of the original D614 mutation in China, and the emergence of G614 mutation (mainly in Europe).

Researchers have been recently interested in tracking mutations that might target the S protein, resulting in a more persistent version of SARS-CoV-2. Using the Global Initiative for sharing All Influenza Data (GISAID), Korber et al. have conducted an analysis pipeline to track the changes and progression of the S protein of SARS-CoV-2, aiming to alert the broader community to changes in the number of genetic alterations that may indicate “positive selection” along with changes in either viral phenotype or antigenicity (Korber et al., 2020). The mutation spike D614G was the first site of urgent concern detected in early March; it was observed 7 times in 183 sampled sequences; four of the first D614G stains were detected in Europe, one in Mexico, one in Brazil, and one in Wuhan (**Figure 4A**). The D614G mutation was followed by another two mutations, one silent mutation (C-to-T mutation in the nsp3 gene) and a C-to-T mutation that leads to an RNA dependent RNA polymerase (RdRp) amino acid modification (Korber et al., 2020). The combination of the three mutations is referred to as “G clade”; and it first appeared in Europe. In mid-march, a second report had revealed that the G clade started to spread and was present in 29% of the samples worldwide; but still found mainly in Europe (Korber et al., 2020). In early April, another sample showed that the frequency of G614 was spiraling; additionally, the G614 version has emerged and became the dominant version in Europe and China in a few weeks (**Figure 4B**). Data regarding the spread of D614 in Africa and South America are still sparsely sampled. Therefore, the D614G mutation might have originated from a single ancestor from lineages that were first found in China. Additionally, three distinct patterns regarding D614G mutations are observed:

1. Mutations that seem to belong to the same lineage (P1263L, in the UK and Australia. Also, A831V, in Iceland)
2. Mutations that are present in various areas and scattered worldwide (L5F)
3. Mutations that belong to the same geographic region; however, they develop in distinct lineages (S943P, present only in Belgium)

These recent findings indicate that SARS-CoV-2 genome has undergone mutations; and D614G is just one of plenty, as Korber et al. have also detected another 13 mutations in the S protein. They additionally identified two reasons that may illustrate why D614G is linked to increased transmission; the first one is the unique structure of D614 and its placement on the S protein promoter; hence it can easily communicate with the nearby promoter (Korber et al., 2020). Remarkably, mutations in the spike protein of SARS-CoV-2 impose conformational modifications, altering the virus antigenicity and affecting the transmissibility, which can account for higher mortalities in some countries (Eaaswarkhanth et al., 2020). The second method that D614 mutation might affect transmission is that D614 is hammered in an immunodominant antigenic determinant in the native S protein of SARS-CoV; this epitope is acknowledged by antibodies found in patients who recovered from the native SARS-CoV. Additionally, Wang et al. stated that this antigen is attacked by a vaccine in primates. Accordingly, this epitope might have acquired resistance to “protective-D614-directed antibody responses” in people who carry the infection; consequently, this emergence of D614 to a G614 version might make those infected patients prone to reinfection. This genetic evolution is crucial in understanding the change imposed by the D614G on antibody responses and Convalescent Plasma Transfusion (CPT) (Korber et al., 2020).

## Genetic/Molecular Predisposition and Underlying Conditions as Risk Factors

### Gender Differences

Various studies have been published that discuss the distribution and prevalence of COVID-19. One of the more interesting observations regarding the virus’s spread is the fact that it seems to be more prevalent in males. One Asian study found that the ACE2 receptor is much more commonly expressed in males than in females (Zhao et al., 2020). Another study that took place in China showed that the gender distribution of the disease in a population of 140 patients was the same while in the critically ill subgroup those affected were mostly males (Zhang J.-J. et al., 2020). Another later report from China that spanned data from 552 hospitals showed that just over half the patients were men (Guan et al., 2020). Finally, another study that examined case data for over 1000 Chinese cases showed that males had an over two-fold higher mortality rate than females with increased severity of the disease (Jin et al., 2020).

Looking at these observations, the data does seem to indicate that there is a predisposition among males for COVID-19. There are several hypotheses that may support the reason for this predisposition, the first one being that smoking may be involved as it is much more common in males than in females (Shah et al., 2019). Of note, a single study showed that ACE2 is normally expressed in the same level among all different ethnicities, genders, and age groups (Smith et al., 2020). However, in the study, smokers seemed to have a much higher expression level of ACE2. This evidence is not sufficient to fully explain the sex predisposition as current literature does not support smoking as a significant predisposing factor for infection with COVID-19. The two studies that were performed in China (Zhang J.-J. et al., 2020; Guan et al., 2020) each had a very low number of smokers among the study population which was disproportionate to the number of smokers within the Chinese population itself. As of now, there is no concrete evidence to support a strong association between smoking and predisposition to COVID-19.

A study performed by Fagone et al. (2020), computational methods such as network analysis were used to elucidate what actually happens within cells once they are infected by the virus. The authors also predicted novel therapeutic agents and repurposed ones that may aid in the battle against the disease. A strong focus was given to comparing the transcriptional signature induced by the viral infection in the infected cells between males and females. The transcriptomic profile of lung tissues from healthy individuals was cross examined with that of tissues infected by the virus. Of note, the transcriptomic profile of women aged 40 to 60 years old was highly similar to the transcriptomic profile of SARS-CoV-2-infected tissue. This similarity was more evident in women’s transcriptomic profiles than men. The authors predicted that this profile leads to a higher threshold for acute response in females of that age. However, it is too early to conclude that this hypothesis is correct. Both a review of epidemiological data, as well as *in vitro* studies are further needed to analyze this relationship.

Female specific hormones that are related to menopause could be the reason for this difference in the transcriptomic profile. The study found that 2 of the COVID-19 induced genes (neutrophil chemotactic factor CXCL1 and the predominantly dendritic cell chemotactic factor CCL20) undergo regulation by androgen receptors. Two other genes were found to be regulated by estrogen receptors (ER). Androgen receptors are implicated in the regulation of the immune system especially in the recruitment of macrophages and neutrophils; two types of cells that are heavily associated with COVID-19 infection (Zhang W. et al., 2020). CXCL1 and CXCL2 have appeared to be heavily involved and modulated by exposure to the COVID-19 virus. This modulation may be different between genders and may impact the outcome of the infection. Furthermore, estrogen receptors are also heavily involved in immunity specifically in the antiviral response even to the point that ER modulators have been suggested as therapeutic drugs. Selective estrogen receptor modulators (SERMs) (such as toremifene and equilin) have been especially suggested as they interfere at the post viral entry step and affect the triggering of fusion between the viral and endosomal membranes (Kovats, 2015).

Another factor that is suspected to be a deterministic one in the observed gender difference in COVID-19 transmission, severity, and mortality is the suspected differences in the expression of the TMPRSS2 (Hoffmann et al., 2020). In order to enter the cell, SARS-CoV-2 adheres to ACE2 receptor and TMPRSS2 for protein priming. TMPRSS2 is profoundly expressed in the kidney, prostate, vesicles, and ducts (Vaarala et al., 2001). In theory, the variation in the expression of TMPRSS2 between males and females may serve as a predisposing factor leading to increased risk of COVID-19-associated mortality in males. However, so far little evidence has been presented to support these claims. A study that analyzed the possibility of seminal transmission of the virus found it to be highly unlikely. Nevertheless, it is worthy to note that 19% of the patients included in the study reported scrotal discomfort as one of their symptoms suggesting potential damage to testicular tissue (Pan et al., 2020).

### Ethnicity

A hypothesis suggesting the role of coagulation having a major effect in the pathogenesis of COVID-19 was further examined to see differences among different races and ethnicities (Fogarty et al., 2020; Zhou F. et al., 2020). Since thrombotic risk differs between races and ethnicities, studies show that African Americans are at higher risk than Caucasians populations (Huang et al., 2019) and the Chinese population has a lower risk of venous thrombo-embolism which is around 3-4 times lower than that in Caucasians (Liao et al., 2014). A study performed in Ireland examining the coagulation in Caucasian patients, the data observed showed a significant relationship between coagulopathy and severity of COVID-19 disease (Fogarty et al., 2020). The coagulation activity was evident with an increase in levels of D-dimer in addition with an increase in lungs fibrinolysis. Therefore, we can relate that races with high risk of thrombosis and the relation found between the pathogenesis of COVID-19 and coagulopathy show the possibility that those races are susceptible to COVID-19 mortality (Fogarty et al., 2020).

In Chicago, as of 11^th^ June 2020, 29.8% of the reported COVID-19 positive cases were African-Americans and 44.7% of the deaths where African-Americans as well (Shah et al., 2020). African-Americans have higher COVID-19 related mortality and morbidity (Krishnan et al., 2020). This can be due to densely populated areas where African-Americans live with low income and poor access to health care facilities (Shah et al., 2020). Moreover, a study examining the relation between increase the in gene expression of ACE 2 and TMPRSS 2 is related to increased symptoms severity in COVID-19, in the meantime African Americans show and increased gene expression of ACE2 and TRPSS2 putting them at a higher risk with COVID-19 infections (Peters et al., 2020).

Furthermore, as per CDC 33% of hospitalized patients are Black while 8% are Hispanic (CDC, 2020a). Death rate is highest in black populations with 92.3 deaths/100,000 population and 74.3 deaths/100,000 population for Hispanics. A few factors may be contributing like those ethnic minorities tend to live in densely populated areas with difficult access to medical care. Most of them work in essential service industries with no paid sick leave and no medical insurance.

### Lower Infection Rate of COVID-19 in Children

Globally, the number of reported children’s COVID-19 cases is significantly lower than adult cases (El-Shabrawi and Hassanin, 2020; Hossny and El-Owaidy, 2020). January 20, 2020, marks the reporting of the first pediatric confirmed case of SARS-CoV-2 infection in Shenzhen, China, which escalated to 20 reported pediatric cases by January 31. Since then, several pediatric cases were announced worldwide. Yet, the pediatric clinical and epidemiological patterns of COVID-19 remain highly unclear (Tezer and Bedir Demirdağ, 2020).

Based on a respiratory infections retrospective study conducted in China on 366 hospitalized children, infection with COVID-19 took place early in the epidemic causing critical respiratory infections (Liu et al., 2020). Most of the pediatric cases were family cluster cases with adult patients’ epidemiological links (Rasmussen et al., 2020). Even though a large number of COVID-19 cases in children are mild with lower reports of cough and fever in comparison to adults, based on similar findings from the Chinese data, serious COVID-19 illness might still take place in this age group leading to hospitalization (CDC COVID-19 Response Team, 2020). Results from the largest Chinese research on positive COVID-19 confirmed cases showed that children aged 2-19 contributed to 2% of the total 44,672 confirmed cases with 0.9% of those cases below the age of 10 years at diagnosis time (Ludvigsson, 2020). Likewise, Italian data published on March reported only 1.2% children’s COVID-19 cases out of the 22,512 positive Italian cases (Ludvigsson, 2020). Moreover, the Korean CDC correspondingly declared, until the 20^th^ of March, 6.3% of all confirmed COVID-19 cases were children below 19 years old, while in the U.S. 5% of the 4,226 reported cases up to March 2020 were children (Ludvigsson, 2020; Brodin, 2020). Based on initial evidences, human susceptibility to COVID-19 paradoxically decreases with increasing the levels of individual’s allergic conditions, such as asthma and allergen sensitivity (Abrams and Szefler, 2020). Furthermore, available evidence proposes an inverse relationship between age and COVID-19 severity, suggesting high vulnerability of infants to COVID-19 mortality (Hagmann, 2020). Although several studies suggest that the majority of children seem to undergo moderate COVID-19 disease symptoms with a recovery period of one to two weeks, those with underlying conditions such as cardiovascular disease, chronic pulmonary disease (including asthma), and immunosuppression are at higher significant risk of more severe symptoms with COVID-19 (Tezer and Bedir Demirdağ, 2020).

Despite the limited data available on the pathogenesis of COVID-19 in children, various large epidemiological studies imply lower severity of the disease manifestation in children than in adults (Brodin, 2020). Current international evidence indicates that children are more likely to have mild COVID-19 symptoms compared to adults. Cough and fever remain the most common symptoms in children who appear to more likely report gastrointestinal symptoms than adults. Mild disease or lack of a clinical presentation may contribute to why most countries detect relatively fewer cases in children, compared to adults (El-Shabrawi and Hassanin, 2020; Hossny and El-Owaidy, 2020).

**Figure 5:**
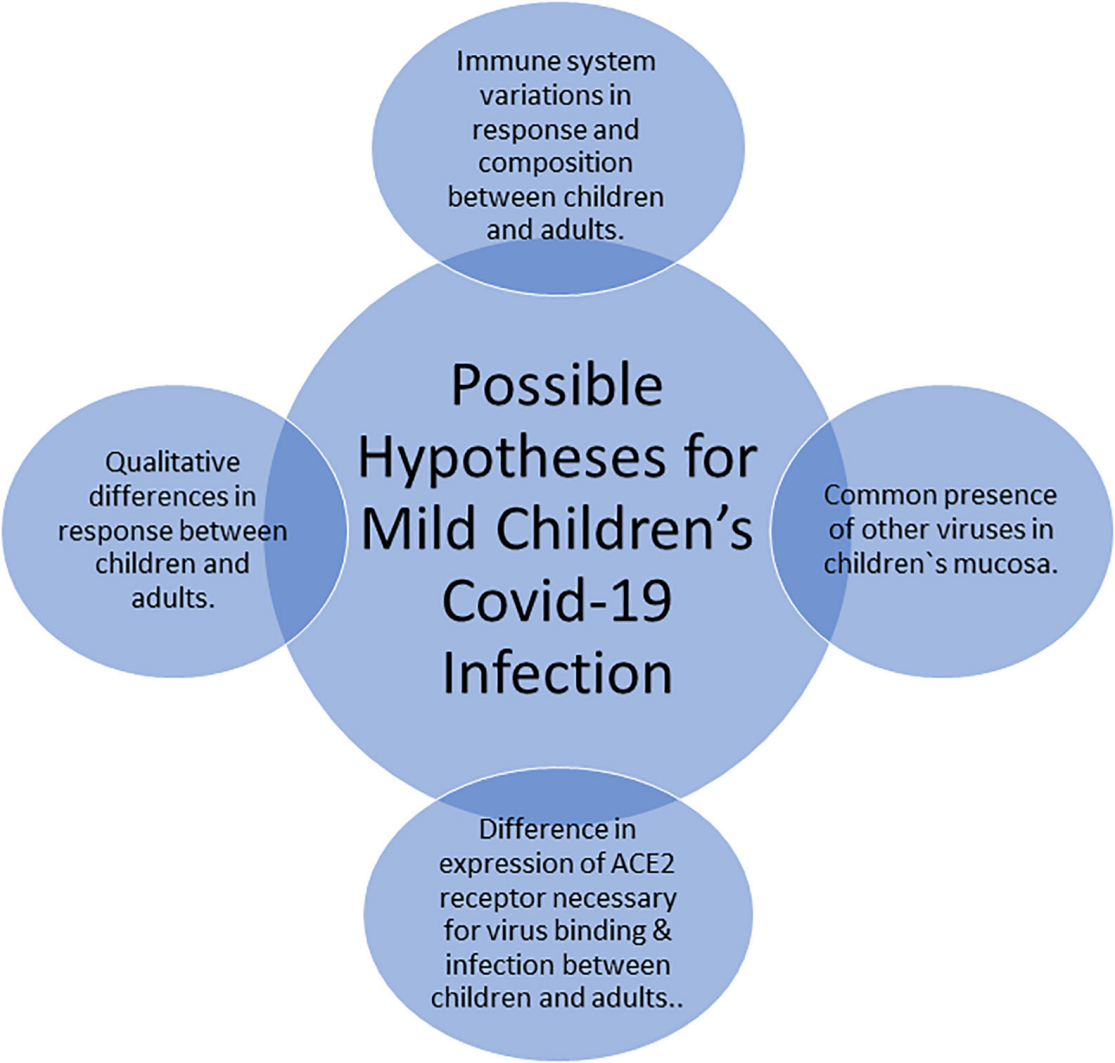
Possible hypotheses for the mildness of COVID-19 infection in children.

The rationale behind the mild children’s COVID-19 infection remains elusive and multiple hypotheses exist as summarized in **Figure 5**. Principally, adults and children immune systems vary from each other with respect to their functional responsiveness and composition. In addition, there are variations between the immune system of teens, pre-school children, and infants. For instance, newborns undergo dramatic changes and get exposed to a wide range of external environmental exposures (Brodin, 2020). Moreover, infants during their first months have some maternal antibodies which are not present in older children. The qualitative difference in response to the COVID-19 virus between children and adults may provide one possible explanation for the less severe COVID-19 disease presentation in children (Brodin, 2020). The familiar presence of several simultaneous viruses in the mucosa of lungs and airways in children, which might reduce the growth of SARS-CoV-2 by direct virus-to-virus competition and interactions, provides also a potential explanation which aligns with the current pandemic data that suggest a possible association between COVID-19 severity and the number of viral copies (Brodin, 2020). Furthermore, another credible theory for the milder children infection with SARS-CoV-2 is the expression variability of the ACE2 receptor, which is mandatory for the binding and infection of the SARS-CoV-2 virus. The ACE2 receptor is not expressed in immune cells but rather in the intestines, airways, and lungs. Treatment of hypertension, which is much common in adults than in children, using ARBs or ACE inhibitors induces expression of ACE2, whose raised level led to the possible explanation to the worse effects of SARS-CoV-2 infection in adults (Brodin, 2020).

Multiple plausible mechanisms exist within the fields of virology, anatomy, and immunology which could explain the mild presentation of COVID-19 infection among children. In COVID-19 pandemic, children seem to be at lower risk of infection than adults (Liu et al., 2020). Due to the international under-representation of children’s COVID-19 testing, it is difficult to make assumptions on different symptom profiles between children and adults and age-specific incidence patterns whose evidence is greatly dependent on testing cases. Consequently, an adequate number of children in a population-based testing strategy and significant samples sizes are needed to explore the true COVID-19 infection among children (Hossny and El-Owaidy, 2020). Reinforcing infection control measures, performing health management and increasing awareness within communities can never be overemphasized to help reduce the global pandemic (Rasmussen et al., 2020). A lot has been learnt about COVID-19 in a very short period of time, yet there is still much to learn about the impact of children on viral spread along with the impact of COVID-19 virus on children (Hagmann, 2020).

### Cardiovascular Diseases and COVID-19

There is an established association between different coronaviruses types and cardiovascular diseases (Xiong et al., 2020). COVID-19 also follows this association. According to the U.S. CDC reports, a person with a co-morbid health condition seems to face more risk from severe COVID-19 than persons without this underlying condition. The percentage of patients having one or more risk factor or underlying disease condition was higher among those requiring emergency unit admission (78%) and those requiring hospitalization without critical care unit admission (71%) than that among the individuals who were not hospitalized (27%). Chronic pulmonary and cardiovascular disease (CVD) and diabetes mellitus were the most recorded cases (CDCMMWR, 2020).

These reports were supported by a lot of studies. In a case series study, 18 cases with comorbidity out of a total of 21 cases admitted to emergency unit in a hospital in Washington were identified (Arentz et al., 2020). Chronic renal disease (47.6%) and congestive heart failure (42.9%) were the most common reported comorbidities. In a larger case series study of 138 hospitalized cases in China (Zhou F. et al., 2020), (14.5%) of the cases had cardiovascular diseases and (31.2%) had hypertension. A retrospective cohort study was done on 191 Chinese cases admitted to the hospital (Zhou F. et al., 2020) from which 48% had an underlying co-morbid disease. Among the most common comorbidities are hypertension (30%) and coronary heart disease (8%).

The cardiac dysfunction occurred has many explanations. One theory of CVD associated risk is explained by the laboratory findings associated with COVID-19 patients. The clinical picture of hospital admitted patients with confirmed COVID-19 was studied (Huang et al., 2020). Laboratory findings showed that levels of troponin-I were increased in 5 (12%) out of 41 patients indicating acute heart injury. This includes 4 (31%) out of 13 patients admitted to emergency unit and 1 (25%) out of 28 patients not admitted to hospital. Although there is an increase in the levels of procalcitonin and proinflammatory cytokines occurring in most patients indicating inflammatory responses, it has been also shown that COVID-19 increases T- helper 2 cytokines (Huang et al., 2020). Nevertheless, the severe effects of COVID-19 on pulmonary tissues and the resulted hypoxemia, perfusion, and vascular abnormalities (Lang et al., 2020) greatly affect the heart function.

Another theory is the drugs which are used in COVID-19 management which may in turn affect heart function. One of the suggested antiviral drugs used is lopinavir/ritonavir. According to the British National Formulary 2020, this combination is used with caution in patients with cardiovascular diseases and several drug-drug interactions occur with other cardiovascular medications (Driggin et al., 2020; Osborne et al., 2020). Remdesivir is another medication which is emergency-use authorized drug (Commissioner O of the, 2020b). In a recent randomized clinical trial, patients receiving remdesivir stopped taking it prematurely due its side effects including worsen the cardiopulmonary status (Wang et al., 2020b). Chloroquine which is another under-investigation medicine for COVID-19 management (Cortegiani et al., 2020) is well known to cause severe arrhythmia (Moore, 2020). **Table 1** shows some of the drugs that are either currently used or under clinical investigations but cause certain cardiovascular adverse effects.

**Table 1:** Potential therapeutic drugs for COVID-19 and their cardiovascular effects.

Medication	Status	CV side effects
Remdesivir	Emergency-use authorization by FDA (Lim et al., 2020).	Worsen the cardiopulmonary status (Wang et al., 2020b).
Chloroquine	On late May 2020, a pause of this treatment arm from the WHO solidarity trial due to safety concerns (“Solidarity” clinical trial for COVID-19 treatments). Mehra et. al. retracted their Lancet paper stating that Chloroquine decreases hospital survival (Mehra et al., 2020). On 3^rd^ June 11, 2020, based on the mortality data, WHO decide to continue this arm of the study (“Solidarity” clinical trial for COVID-19 treatments)	Severe arrhythmia (Moore, 2020).
Lopinavir/ritonavir (Shaffer, 2020)	Under-investigation by clinical trials (WHO solidarity trial) (“Solidarity” clinical trial for COVID-19 treatments).	Causes dyslipidemia and used with caution in patients with CVD (Dorward and Gbinigie, 2020)
Corticosteroids (Shaffer, 2020)	Under-investigation by clinical trials as a support agent (Huanzhong, 2020) but no clinical evidence of their efficacy until now(ASHP-COVID-19-Evidence-Table.pdf).	may lead to edema or fluid retention and may cause hypertension, and arrhythmias (Yasir et al., 2020).

These data show that many of the drugs that are either used currently or that are potential candidates for COVID-19 management, have cardiovascular adverse effects. Most of these drugs are still under clinical trials and some of them are lacking a clinical evidence for their use in COVID-19 patients. In addition, the use of some of these drugs has been a subject of debate, specifically, oseltamivir and chloroquine. Oseltamivir has been widely consumed by COVID-19 patients although there is no evidence to support its use. Mehra et al. in their Lancet paper claimed that chloroquine decreases hospital survival, subsequently WHO temporarily suspended the use of chloroquine for the treatment of COVID-19 patients. Eventually, this paper was retracted, and the WHO reverted back to test this drug in its clinical trials. These contradictory results stemmed a wide range of unwanted disputes, especially during a pandemic, adding more perplexity to an existing tensed situation.

Not only the medications used in COVID-19 management is supposed to affect the cardiovascular status, but also the medications used by CVD patients may affect the COVID-19 susceptibility. For example using different ACE inhibitors or ARBs may cause upregulation of ACE2 receptors (Wang et al., 2016; Kai and Kai, 2020) and it is well established that ACE2 is a functional receptor used for entry of the COVID-19 to the cell, and it is supposed that this upregulation worsens the impact of infection (Nadar et al., 2020; Zhou P. et al., 2020). Another study supposed that COVID-19 enter the cell via ACE2 receptors but then downregulates it, facilitating neutrophils infiltration, lung injury, and attenuating the cardio protection (Vaduganathan et al., 2020). Recent studies suggest that practically, there is no association between ACE inhibitors/ARB use and testing positive for COVID-19, and that using these drugs has no effect on expanding the danger of COVID-19 hospital admission, including lethal cases and those admitted to emergency units (Abajo et al., 2020; Mehta et al., 2020; Reynolds et al., 2020). There is a lot of contradictory results about the actual reflection of using these drugs in COVID-19 management and further studies are needed to provide a clinical evidence (Elimination or Prolongation of ACE Inhibitors and ARB in Coronavirus Disease 2019 - Full Text View; Fosbøl et al., 2020). Several international heart associations recommend patients already using ACE inhibitors/ARBs and are infected with COVID-19 to continue using their medication (ACEI/ARB Use in COVID-19 Patients With Hypertension; Czarska-Thorley, 2020). Physical distancing and quarantine as a measure to limit the spread of the current pandemic and the subsequent changes in the population lifestyle may also influence the cardiovascular status of these patients. This may be due to less interaction and less positive relationship with other people or due to lower physical activities (Jiménez-Pavón et al., 2020; Guzik et al., 2020).

## Environmental and Occupational Risk Factors

### Different Occupations That Are Most Vulnerable to COVID-19 Infection

After COVID-19 was declared as a pandemic, a widespread concern has been raised for the occupations that have the highest risk of catching the infection. There is a belief that work-related exposure contributes to the high infection rate of COVID-19 (Koh, 2020) and it should be classified as an occupational illness (Godderis et al., 2020). In fact, the beginning of the pandemic spread was in a workplace, the fish market in Wuhan, where more than half of the cases (55 %) originated from that market, compared with only 8.6 % of the cases were related to it after shutting down (Li Q. et al., 2020). In Singapore, some of the first cases were linked to work meetings, where local and international employees met, resulting in three infected employees from Singapore, one from the UK, one from Malaysia and two others from South Korea. The international cases were not detected until after leaving the country (Koh, 2020), which might be a source of spreading in the other countries. In addition to the previous cases, occupational exposure was most probably the cause of other earlier cases in Singapore, such as a tour guide, workers in a jewelry store and a retail store who served Chinese tourists, a taxi driver, workers in construction site and others (Koh, 2020). The fear increased after looking at the numbers of infected people during past pandemics due to dealing with infected co-workers. It was estimated that after the H1N1 pandemic, approximately 8 million workers were infected and went to work, and this might have led to infecting another 7 millions of their colleagues (Drago and Miller, 2010).

Recently, a study has been conducted to estimate the frequency of workers’ exposure to infectious diseases in the U.S. to help in containing this pandemic and any other future outbreak. The study revealed that the health care workers were the highest in the exposure frequency (Baker et al., 2020). The actual data supports that the risk of infection due to occupation is the highest among frontline health care providers. A Chinese study stated that till the 3^rd^ of February of 2020, 29% of the patients in Wuhan hospitals were health care workers who had been infected while performing their duties. It was assumed that a patient in the intensive care unit infected at least 10 workers (Wang D. et al., 2020). The basic reproductive number (R_0_) was suggested to be 2.2, which means that one infected person can infect 2.2 others, and this implies a fast rate of person-to-person transmission (Li Q. et al., 2020; Wang D. et al., 2020). In Italy, from February 20^th^ till nearly mid-March of 2020, it was reported that the number of infected health care professionals was 350 (about 20%) and some of them died (Remuzzi and Remuzzi, 2020), while at the end of March 2020, it reached 5000 infected workers and 40 deaths (Burdorf et al., 2020). An article in the UK indicated that during health staff screening for SARS-CoV-2, 5 % of them were positive on the 10^th^ and 11^th^ of March of 2020 and this percentage increased to be 20 % at the end of the month. However, the article suggests the hospital-acquired transmission of Covid-19 to the workers from patients was not a significant way of the spread (Hunter et al., 2020). it is reported that 9% of all COVID-19 cases in Italy are health care workers (Fusco et al., 2020). Looking back at previous epidemics proves that the health care workers were hugely affected. For instance, the health workers were one-fifth of the total worldwide cases during the SARS epidemic (Chan-Yeung, 2004). It is plausible to have a high proportion of infected health providers due to dealing with numerous infected individuals and being exposed to high viral loads (Burdorf et al., 2020). With the shortage of the personal protective equipment (PPE), the number of infected and dead health care workers can massively increase, as the case in Italy (Ranney et al., 2020).

There are other occupations that are vulnerable to COVID-19 infection. According to a U.S. study, the Protective Service personnel and Personal Care and Service personnel were among the highest groups in occupational exposure to infection, followed by Community and Social Services personnel (Baker et al., 2020). This is in agreement with an article suggesting that employees with public-facing roles have a high chance to get infected, for instance, policemen, firefighters, educational service workers, childcare workers, public transportation drivers and a few others (Sim, 2020). In addition, a high rate of infection can be within workers that have to interact with a large number of the public, for example shop or restaurant workers in addition to delivery workers. Furthermore, the jobs that need close physical proximity with others are at a high risk, as physiotherapists, nail technicians, and hairdressers (Burdorf et al., 2020). Another group was highlighted, the ship and airline crews (Sim, 2020). The number of incidences on a cruise ship where approximately 1.9 % of its staff (20 workers of total 1068) were confirmed as positive cases after one week of detecting the first case among the crew, is a support for the previous opinion (Kakimoto et al., 2020).

The consequences of COVID-19 pandemic on workers can be physical and economical. The health effect may be related to the virus itself or due to stress (Sim, 2020). For example, increasing the percentage of infected health care providers, increases the work burden on the healthy ones, which leads to high stress, either from the work over-load, or the fear of getting the disease, or transmitting it to their families, or from the quarantine after dealing with infected patients or coworkers (Sim, 2020; Xiang et al., 2020). The health workers are not the only workers that can suffer from mental illness in the pandemic, others can be stressed from the quarantine or the idea of losing or have already lost their jobs (Sim, 2020). Some will stop going to work because of the contagion, and some will be forced to work while being sick. High proportion of absenteeism or presenteeism may raise the stress or worsen the condition of ill workers or infect others and reduce the work productivity (Kinman, 2019; OSHA, 2020). Beside the health issues, many businesses have been closed, resulting in workforce reduction. This causes huge impacts on employees, especially day laborers (Sim, 2020). Unfortunately, some workers do not have the luxury to work from home. On the other hand, there are thoughts that the pandemic has beneficial effects on the workplaces. The employers will have to put safety measures to protect their employees and using distance meeting more often will result in reducing traffic and decreasing the air pollution resulting from the fuel consumption (Sim, 2020).

As a conclusion, to keep the wheel turning, precaution should be taken to protect our workers, especially the health care workers, as we cannot afford losing them in this crucial time. The Occupational Safety and Health Administration (OSHA) in the U.S. advises to plan how to provide a safe work environment to keep the workers safe. It provided instructions to be followed and classified the occupational risks according to the exposure risk to use the resources effectively (OSHA, 2020). Presently, it is important to provide support to workers, socially and mentally (Koh, 2020). Adequate sick leave regulations are a significant need to reduce the stress among workers (Kinman, 2019; OSHA, 2020). A plan and training should be set before ending the lockdown to prevent losing more workforce (Agius, 2020).

### Vaccination Status

Bacillus Calmette–Guérin (BCG) vaccinations,against tuberculosis, might be affecting COVID-19 mortality in countries where BCG vaccine is still mandatory (Miyasaka, 2020). Recent data shown in **Table 2** below shows deaths rates in different countries and vaccination for BCG with different strains. As shown in **Table 2**, countries that do not give BCG vaccination or even stopped giving it have higher mortality in comparison with countries still on BCG vaccination.

**Table 2:** COVID-19 deaths per million population and BCG vaccination.

Country	Availability of BCG Vaccination (b)	Strain for BCG (b)	Deaths per 1 Million (a)
Taiwan	Still available	Japan	0.3
Iraq	Still available	Japan	2
China	Still available	Russia/Bulgaria	3
Japan	Still available	Japan	4
Australia	1950-mid 1980s	Connaught	4
Korea	Still available	Different strains	5
Norway	Unknown to 2009	Denmark	39
Turkey	Still available	India	40
Finland	1941–2006	Denmark	43
Iran	Still available	Denmark	75
Germany	1961–1998	Pasteur	82
USA	Not used	NA	207
Sweden	1940–1975	Denmark	274
France	1950–2007	Denmark	381
UK	1953–2005	Denmark	419
Italy	Not used	NA	478
Spain	1965–1981	Denmark	540

^a^Obtained from Worldometer (Coronavirus Update (Live): 4,714,317 Cases and 312,298 Deaths from COVID-19 Virus Pandemic).

^b^Obtained from Ritz and Curtis (2009) and Zwerling et al. (2011) (The BCG World Atlas: A Database of Global BCG Vaccination Policies and Practices; Sharma et al., 2020).

BCG vaccine has a heterologous effect on the immunity as it protects against various pathogens probably by inducing increased immune response towards infections (Hirve et al., 2012). Also, different BCG strains used show different mortality rates as seen in **Table 2**. That is BCG strains from Japan & Russia show lower COVID-19 mortality, which are both early strains while BCG strains from Denmark show higher mortality rates in COVID-19 patients (Miyasaka, 2020). This may be due that early BCG strains can stimulate trained immune responses due to their strain richness. However, data from Australia and Finland is opposite to that since they ceased BCG vaccinations years ago, yet their mortality rate is also low. So definitely BCG vaccine is not the only factor in minimizing COVID-19 mortality but possibly since in both Finland and Australia social distancing is effective as both countries have small population densities in addition to their excellent health care facilities.

Italy has an old population representing 23.3 % with ages above 65 years old as per United Nations, World Population Prospects 2019 (Dowd et al., 2020). Also, Italian families tend to live in near vicinity areas with close contact between different generations (Kalmijn and Saraceno, 2008). Therefore, the frequent contacts between different generations and close living proximity resulted in speeding Covid-19 outbreak in Italy (Mossong et al., 2008). So its advised that old population rich countries need to have tough measures for protection to help flatten their curve and reduce the health care load in order to prevent high rates of death (Dowd et al., 2020).

In summary, we can see that old populations living in denser neighborhoods with low socio-economic class and poor access to medical care are more at risk to COVID-19. Other factors like BCG vaccination and certain enzymes gene expression may be contributing to COVID-19 pathogenesis yet more research needs to confirm this.

### Pregnancy and COVID-19

There is a concern about pregnant women and neonates of being at high risk to get COVID-19. Although several studies were published in the last months about COVID-19, there are still limited data available to indicate the risk of infection of this population (Novel Coronavirus 2019 (COVID-19)). According to CDC recent reports, pregnant women with COVID-19 have higher risk of severity of illness like ICU admission and mechanical ventilation in addition to increasing the risk of premature birth. No evidence until now of having higher mortality. The overall risk of infection in this category are still low and significantly lower than other respiratory tract infection in this category (Novel Coronavirus 2019 (COVID-19); CDC, 2020b).

According to the available data, the type of delivery, vaginal or cesarean, has no effect on the viral transmission (Karimi-Zarchi et al., 2020; Ryan et al., 2020). Moreover, there is no evidence that COVID-19 can cause congenital abnormalities or miscarriages (ECDC, 2020). In the beginning of the pandemic, there was no evidence of vertical transmission, but now there is a suggestion of its presence (ECDC, 2020; Patanè et al., 2020; Fornari, 2020). A study showed that 50% of neonates born to mothers with COVID-19 were cured and discharged, 40 % were hospitalized and 10% (1 case) died, concluding that perinatal COVID-19 infection can lead to severe adverse effects (Zhu et al., 2020), while other studies revealed that the newborns were well and discharged (Chen S. et al., 2020; COVID-19 virus infection and pregnancy; Occupational health advice for employers and pregnant women during the COVID-19 pandemic, 2020; Wang X. et al., 2020). Most health institutions recommend breastfeeding, if the mother has mild symptoms of COVID-19 or asymptomatic and able to feed her baby, with taking infection control precautions, such as wearing facemask while breastfeeding, proper handwashing, the baby’s bed should be placed 2 meters away from the mother’s (Davanzo et al., 2020). If the mother does not have the ability to feed her baby, it is advisable to use expressed breastmilk and avoid the frequent breastmilk substitutes use (Davanzo et al., 2020).

### Mask Wearing Culture

In response to the COVID-19 outbreak, different approaches regarding mask wearing were raised. A study was applied to study the efficacy of wearing face masks in lowering respiratory viruses’ transmission, including coronavirus, by detecting the virus in both respiratory droplets and aerosols. For people not wearing face masks, The coronavirus was detected in the respiratory droplets of 30 % of the participants and in the aerosols of 40% of them while in people who wear the mask, 0 % was detected in both aerosol and respiratory droplets of the participants (Leung N. H. L. et al., 2020). Wearing face masks is very important as there is some evidence that patients infected with COVID-19 even though they are asymptomatic can spread infection (Bai et al., 2020).

The WHO claims that wearing a mask for everyone may give a wrong sensation of safety which will lead to neglecting important medications, it will also cause shortage of these important masks for the health care workers, this encourages the rational use of face masks (World Health Organization, 2020). The CDC puts a guidance on protecting yourselves and others from the infection (CDC, 2020a), among these rules is that everyone should wear a face cloth at least covering the nose and the mouth when it comes to contact with others keeping in mind that you still need to be socially distant from others by around 6 ft./~2 m. Health authorities need to guide the population about proper use of face masks and also need to think about solutions for the global shortage of masks (Leung C. C. et al., 2020).

In a sample of high socioeconomic status Chinese people, 98% of them were wearing masks when going out (Zhong et al., 2020). In another study in Hong Kong, 94.8 % were wearing masks but from them 13 % wore them in a wrong way (Tam et al., 2020), other regions in China and also some regions in Japan and Thailand choose to make another disposable alternatives (Feng et al., 2020). South Korea, Canada, and Czech Republic guide their Citizens to use masks in public areas (Cheng et al., 2020). In Singapore, the health authority states that there is no need to wear masks if you are well (Wang C. et al., 2020). One approach is done to make a cloth mask for low income countries to be allowed only in case of unavailability of surgical masks (Sugrue et al., 2020). On the other hand, some researchers suggest trying the fiber masks impregnated with antiviral nanomaterial to work in a synergistic way to reduce the infection (Livingston et al., 2020; Sportelli et al., 2020).

The European centers for disease prevention and control recommends wearing masks in pandemics when the percentage of infected people with no symptoms is high especially in closed busy places and for people at high risk or their occupations require them to deal with many people (ECDC, 2020). Wearing the facemasks for certain populations maybe unfavorable. For example, it is advisable by CDC that face masks ought not be utilized by kids younger than 2 or the individuals who may think that it is hard to control them effectively. For instance, essential age youngsters unassisted, or those with respiratory conditions (CDC, 2020c). Wearing a mask for those having a respiratory condition is not suggested as this may make breathing increasingly troublesome (COVID-19 and lung disease Q&A; COVID-19 Frequently Asked Questions). Moreover, patients with shortness of breath are not encouraged to utilize a facemask for expanded period since it can cause them to feel hot and fall apart the brevity of breath (Ren et al., 2016). The breathing difficulty during wearing a mask affects individuals practicing sports and exercising. In these populations finding the best mask for the patient is crucial. For example, a few covers are more breathable than others. Patients can simply maintain a deep breathing and avoid shallow breathing, which can prompt hyperventilation. On the other hand, patients can discover zones that are less populated, making the cover important just when getting through a crowd (Nyenhuis et al., 2020).

In all situations, it is very important for health authorities and policy makers to put a guidance on the rational use of face masks based on the pros and cons mentioned and to encourage initiatives and researches on disposable protecting alternatives or re-use alternatives to help in decreasing as much as possible the vulnerability to COVID-19 infection during this pandemic (Islam et al., 2020; Rubio-Romero et al., 2020).

## Conclusion

Studies of the genomic characterization of COVID-19 show that the virus has much more evolved than its predecessor (SARS-CoV-1). SARS-CoV-2 binds the ACE2 with much higher affinity that SARS-CoV-1, tolerates higher temperatures and is generally more solvable (He et al., 2020). Emerging mutations in the viral genome may affect the transmission and virulence of the virus as well as the susceptibility of certain groups (Korber et al., 2020). Current evidence shows that there are various disparities in the infection and severity of COVID-19 transmission among different groups.

Important differences include variation among different age groups with heightened infection and mortality rates in older ages (Onder et al., 2020) and significantly lowered infection rates among children (Tezer and Bedir Demirdağ, 2020). Differences in infection and mortality can also be observed in different ethnicities such as African-Americans in the U.S. (Fogarty et al., 2020). The presence of underlying conditions especially those of cardiovascular nature is seen to exacerbate COVID-19 symptoms and groups with such conditions show heightened mortality and morbidity (Zhou F. et al., 2020). The three observations are hypothesized to be caused by variations in the expression of either ACE2, TMPRSS2 or both. The dysregulation of ACE2 has been found to be associated with fluctuations in COVID-19 morbidity and the serine-protease TMPRSS2 has been found to be needed in the priming of the virus in preparation to its entry into the host cell (Hoffmann et al., 2020).

As for environmental and occupational exposure, various occupations show increased risk of contracting COVID-19 specifically those involving front-line health care providers and occupations that require face-to-face interaction (Koh, 2020). One of the interesting remarks regard regarding the pandemic is that countries with a vaccination plan that includes the BCG vaccine seem to have a reduced infection and mortality rate (Miyasaka, 2020). There are conflicting opinions regarding the effect of wearing masks on curbing the spread of COVID-19. While the WHO’s policy recommends against wearing face masks (World Health Organization, 2020), the CDC strongly recommends wearing them. The CDC not only recommends wearing face masks, but it also suggests the use of homemade cloth masks as an alternative to surgical masks where those are not accessible (CDC, 2020a).

It is evident that there is a myriad of factors that affect the transmission, virulence, and mortality rates of COVID-19 infection. With the current lack of treatment options and prophylactic measures, more interest should be given to elucidating what makes certain groups at higher risk of COVID-19 complications than others. An aspect that should be given a strong focus is the importance of the molecular landscape of COVID-19 in the determination of the case prognosis. Most importantly the identification of environmental and occupational risk factors is of utmost importance. Finally, close monitoring of the accumulating mutations in the viral genome is a must, both for anticipating response to vaccines/therapeutics as well as predicting changes in susceptibility of vulnerable groups.

## Author Contributions

HA and AA designed the outline of the review. BS edited and proofread the review. All authors contributed to the article and approved the submitted version.

## Funding

This work is supported by an AUC COVID-19 Pandemic Research & Innovation Initiative Grant to AA.

## Conflict of Interest

The authors declare that the research was conducted in the absence of any commercial or financial relationships that could be construed as a potential conflict of interest.

## Abbreviations

SARS, severe acute respiratory syndrome; ARDS, acute respiratory distress syndrome; *α*-CoVs, *Alphacoronavirus*; *β*-CoVs, *Betacoronavirus*; γ*-*CoVs, *Deltacoronavirus*; *δ-*CoVs, *Gammacoronavirus*; CoVs, coronaviruses; SARS-CoV, severe acute respiratory syndrome-related coronavirus; MERS-CoV, Middle East respiratory syndrome-related coronavirus; WHO, World Health Organization; FDA, Food and Drug Administration; EUA, Emergency Use Authorization; IVCC, International Virus Classification Commission; ACE, angiotensin-converting enzyme; MD, molecular dynamics; nsps, non-structural proteins; PCR, polymerase chain reaction; ADE, antibody-dependent enhancement; ARDS, acute respiratory distress syndrome; TMPRSS2, transmembrane protease, serine 2 enzyme; SNPs, single nucleotide polymorphisms; RBD, receptor-binding domain; CXCL, neutrophil chemotactic factor; CCL20, predominantly dendritic cell chemotactic factor; ER, estrogen receptors; HDOCK, hybrid protein-protein docking algorithm; RAAS, renin–angiotensin–aldosterone system; GISAID, Global Initiative for sharing All Influenza Data; RdRp, RNA dependent RNA polymerase; cryo-EM, cryogenic electron microscopy; CPT, convalescent plasma transfusion; SERMs, selective estrogen receptor modulators; CVD, cardiovascular disease; PPE, personal protective equipment; OSHA, Occupational Safety and Health Administration; BCG, Bacillus Calmette–Guérin; ICU, intensive care unit.
